# Mental health at work: a practical framework for employers

**DOI:** 10.3389/fpubh.2025.1552981

**Published:** 2025-04-25

**Authors:** David W. Ballard, Grace C. Lodge, Kathleen M. Pike

**Affiliations:** ^1^One Mind at Work, Rutherford, CA, United States; ^2^Mental Health and Work Design Lab, Columbia University, New York, NY, United States

**Keywords:** occupational mental health, worker wellbeing, performance, mental health, work design, workplace interventions, framework, best practices

## Abstract

Mental health is a universal issue critical not only to individual health and well-being, but also to workforce sustainability and organizational performance. Leaders increasingly understand the imperative to support workers’ mental health but are often unsure of where to start or how to prioritize their actions. Numerous guidance documents for employers have emerged in recent years, but without conducting their own needs and risk assessment and evaluating the policies and practices they have in place, employers are left unaware of specific risks their workers face, effectiveness of their current practices, and actions they could take that would have the greatest positive impact. To address this gap, Columbia University’s Mental Health + Work Design Lab, mental health non-profit One Mind at Work, and corporate ethical standards measurement company Ethisphere partnered to develop a comprehensive framework and corresponding self-assessment to help employers evaluate their organizational-level workforce mental health efforts and strategically invest in evidence-based practices. In this article, we detail the range of organizational-level practices necessary to effectively support and advance workforce mental health and present a framework for assessing and improving these efforts.

## 1 Background

Mental health has historically been a taboo subject in the workplace, despite the role it plays when it comes to both worker well-being and organizational performance. The lack of attention from employers is a major oversight given the prevalence of mental health issues and their potential impact at work. Approximately 1 in 5 adults experience some degree of a mental health concern each year (1) and in 2019, an estimated 970 million people globally had a diagnosable mental health disorder, including 15% of working-age adults (2).

Work itself can also affect mental health. On the heels of the pandemic, 57% of U.S. workers said they experience negative mental health impacts related to work stress and for nearly half (48%) that stress is chronic (3). An extensive body of research links work stress to a variety of negative mental health outcomes (4, 5), including adverse effects on cognitive, emotional, social, and physical well-being. As a result, workers may experience hindered productivity or performance, lessened ability to work safely, or difficulty acquiring or maintaining work (6). Although employers spend far more to address physical health conditions than they do on mental health (7), when performance and productivity decrements are considered, the cost burden of mental disorders far exceeds that of other illnesses including heart disease and cancer (8). Even when the costs of mental health benefits and care are not subsidized by an employer, the indirect costs of poor mental health can be sizable in terms of absenteeism, turnover, presenteeism, and disability (6, 9, 10).

While it is critical to address the economic costs of poor mental health, research has also linked good mental health to job performance (11, 12), so it is equally important to consider the positive benefits of psychological well-being (13). Workforce mental health has been linked to a variety of workplace outcomes for both employees and employers, including job satisfaction, engagement, work performance, and retention (14–17).

Organizational leaders increasingly understand the imperative to support workers’ mental health but are often unsure of where to start or how to prioritize their actions. To date, much of the emphasis has focused on either providing specific benefits that help individual workers manage mental health problems when they arise, or offering resources intended to increase mental health literacy and encourage workers to adopt coping skills that make them more resilient in the face of challenges. While these individual-focused actions may provide useful support, they are unlikely to yield optimal results in the absence of more systemic efforts (18, 19). Real change comes from organizations taking a strategic approach to workforce mental health and investing in comprehensive and systemic improvements.

To this end, numerous guidance documents for employers have emerged in recent years, including ISO 45003, the first international standard on psychological health and safety at work (20), the World Health Organization Guidelines on Mental Health at Work (6), the U.S. Surgeon General’s Framework for Workplace Mental Health and Well-Being (21) and the Framework for Mentally Healthy Workplaces (22, 23). Such guidance provides useful information about addressing workforce mental health, but without conducting their own needs and risk assessment and evaluating the policies and practices they have in place, employers are left unaware of specific risks their workers face, gaps that exist in their efforts, effectiveness of their current practices, and actions they could take that would have the greatest positive impact.

Drawing on the research literature, as well as guidance and standards from global bodies, Columbia University’s Mental Health + Work Design Lab, mental health non-profit One Mind at Work, and corporate ethical standards measurement company Ethisphere partnered to develop a practical framework and a corresponding assessment tool to help employers evaluate their organizational-level workforce mental health efforts and strategically invest in evidence-based practices (24). The resulting Mental Health at Work Index™^1^ is a standardized assessment of organizational-level initiatives to address workforce mental health that employs a maturity model approach (25), enabling organizations to self-assess their program development and pinpoint areas for improvement.

To develop the assessment, the collaborators assembled a core scientific and expert team that was first tasked with organizing the numerous employer practices that can support workers’ mental health and the best practice guidance from various sources into a comprehensive framework. This article details the development process and describes a framework that includes both a continuum for addressing the full range of workers’ needs and 10 categories of evidence-informed practices that support and advance workforce mental health. We conclude with a discussion of how this framework guides the Mental Health at Work Index assessment, future directions for the Index, current limitations, and gaps in both research and practice that need to be closed in order to improve workforce mental health efforts moving forward.

## 2 Methods

Development of the Mental Health at Work Index Framework was led by experts in organizational, clinical, and occupational health psychology, public health, workplace health promotion, corporate compliance and risk management, measurement and evaluation, and management systems. This interdisciplinary approach was intended to span a range of fields related to workforce mental health, integrate typically-siloed research and practices areas, include a diversity of perspectives, and promote collaborative, systems-based solutions.

Literature searches using electronic databases including PsycInfo, PubMed, and Google Scholar focused on both peer-reviewed journal articles and academic texts from 1990 to 2024. Keywords and search terms were based on workforce mental health (e.g., workplace mental health, depression, Employee Assistance Program, behavioral health benefits), workplace health promotion (e.g., worker well-being, health risk assessment, culture of health, workplace wellness), human resource policies and practices (e.g., flexible work, training and development, employee recognition, diversity, equity, and inclusion), occupational stress (e.g., autonomy, job demands, leadership support, incivility), and organizational performance (e.g., turnover, job satisfaction, absenteeism, engagement).

Additional resources included guidance documents and assessment tools from government and NGO sources, such as ISO (20), WHO (6), ILO (26), the UK Health and Safety Executive (27), the U.S. Centers for Disease Control and Prevention (28), the Mental Health Commission of Canada (29), NIOSH (30), the Sustainability Accounting Standards Board (31), the Office of the U.S. Surgeon General (21), and Safe Work Australia (32).

Three senior-level subject matter experts independently categorized identified practices based on key characteristics. Results were then aggregated and consensus built through multiple rounds of live discussion to address agreement/disagreement. The priority at this stage of framework development was broad stakeholder engagement to ensure the framework and related assessment tool represented the issues critical to those who would ultimately apply the framework and use the tool to evaluate and improve workforce mental health efforts in their organizations. To bolster the practical application and utility of the framework and related assessment, a global council of employers provided input and feedback at every stage of a non-quantitative, generative development process that focused on exploring and understanding the various elements of comprehensive, systems-based approaches. This guidance was used to address gaps, provide clarity, reduce redundancy, and identify potential barriers to implementation.

The resulting framework is composed of three levels of intervention that span 10 categories of employer practices that advance and support workforce mental health. While research on comprehensive sets of organizational mental health practices is still in the early stages of quantitative development, this foundational work lays the groundwork for additional data collection. As the field evolves, it will enable the use of more advanced quantitative methods, such as factor and path analyses, to better understand how different practices interact with and relate to one another in real-world organizational settings, as well as any hierarchical and/or sequencing relationships in implementation.

## 3 Levels of intervention

**Figure 1:**
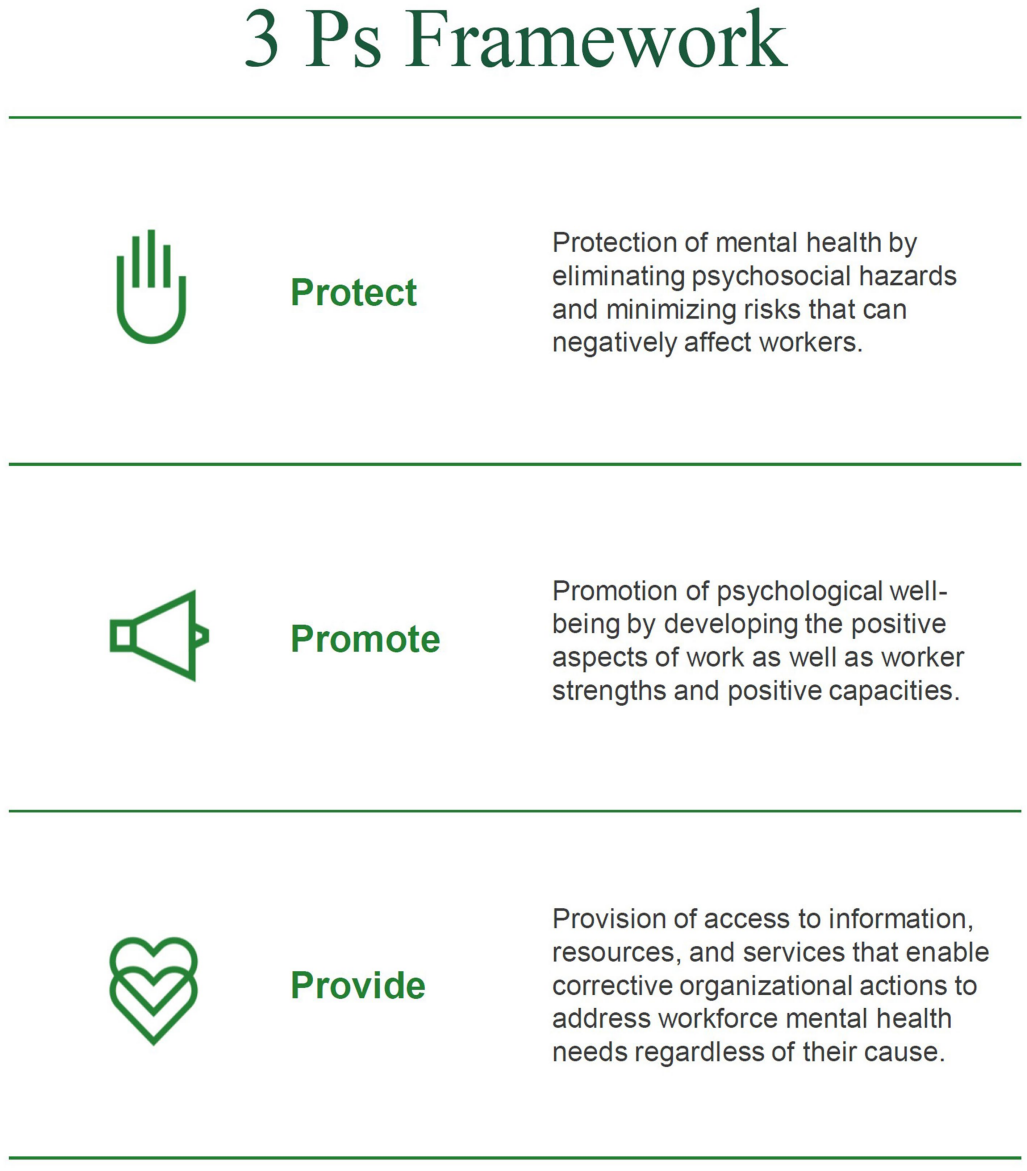
The mental health at work index 3 Ps continuum of protection, promotion, and provision.

Effective workforce mental health efforts address the full range of workers’ needs. Informed by LaMontagne et al.’s integrated approach to workplace mental health (33), the Mental Health at Work Index Framework applies a “3 Ps” continuum across organizational practices. The 3 Ps span: protection of mental health by eliminating psychosocial hazards and minimizing risks that can negatively affect workers; promotion of psychological well-being by developing the positive aspects of work as well as worker strengths and positive capacities; and provision of access to information, resources, and services that enable corrective actions to address mental health needs (Figure 1).

### 3.1 Protection

Protecting workers’ mental health requires going upstream of potential problems and, to the extent possible, preventing them from occurring in the first place. Protection efforts are anchored in the fields of public health, occupational health and safety, and occupational health psychology (33). Using risk assessment and management processes, employers identify hazards, determine the level of risk they pose (e.g., by considering the prevalence, likelihood, and frequency of exposure to the hazards and the severity of impact that would occur), and take steps to eliminate the hazards or apply controls to mitigate negative outcomes (34). In addition to having a system for assessing and addressing psychosocial risks, other protective actions employers can take include education and training to reduce mental health stigma, communications that provide guidance on redesigning work to minimize risk exposure, and policies for preventing bullying, harassment, and discrimination.

### 3.2 Promotion

Mental health exists on a continuum, with the dimension of well-being representing a positive state rather than simply an absence of illness (35). Employer actions designed to promote psychological well-being have foundations in management/leadership, organizational development, and positive psychology and take a multi-level approach with organizational, team, and individual level interventions (23, 33). Practices that promote the positive include initiatives to build psychological safety, training in supportive supervision skills, opportunities to strengthen relationships and a sense of belonging, healthy work design, and wellness programming such as mindfulness, meditation, and self-care.

### 3.3 Provision

Given the prevalence of mental health issues, a portion of the workforce will be experiencing challenges at any given point in time that can include subclinical symptoms, acute conditions, chronic disorders, and crises. Employers can help ensure that workers have access to the information, resources, and services they need to address and manage these concerns. Provision of mental health care is grounded in the work of psychology, psychiatry, and occupational medicine (33) and includes facilitating access to high-quality mental health treatment, short-term counseling for issues affecting work performance, training to help managers identify signs that an employee may be struggling and direct them to available resources, and return-to-work plans to support people following a mental health leave of absence.

## 4 Organizational-level workforce mental health practices

**Figure 2:**
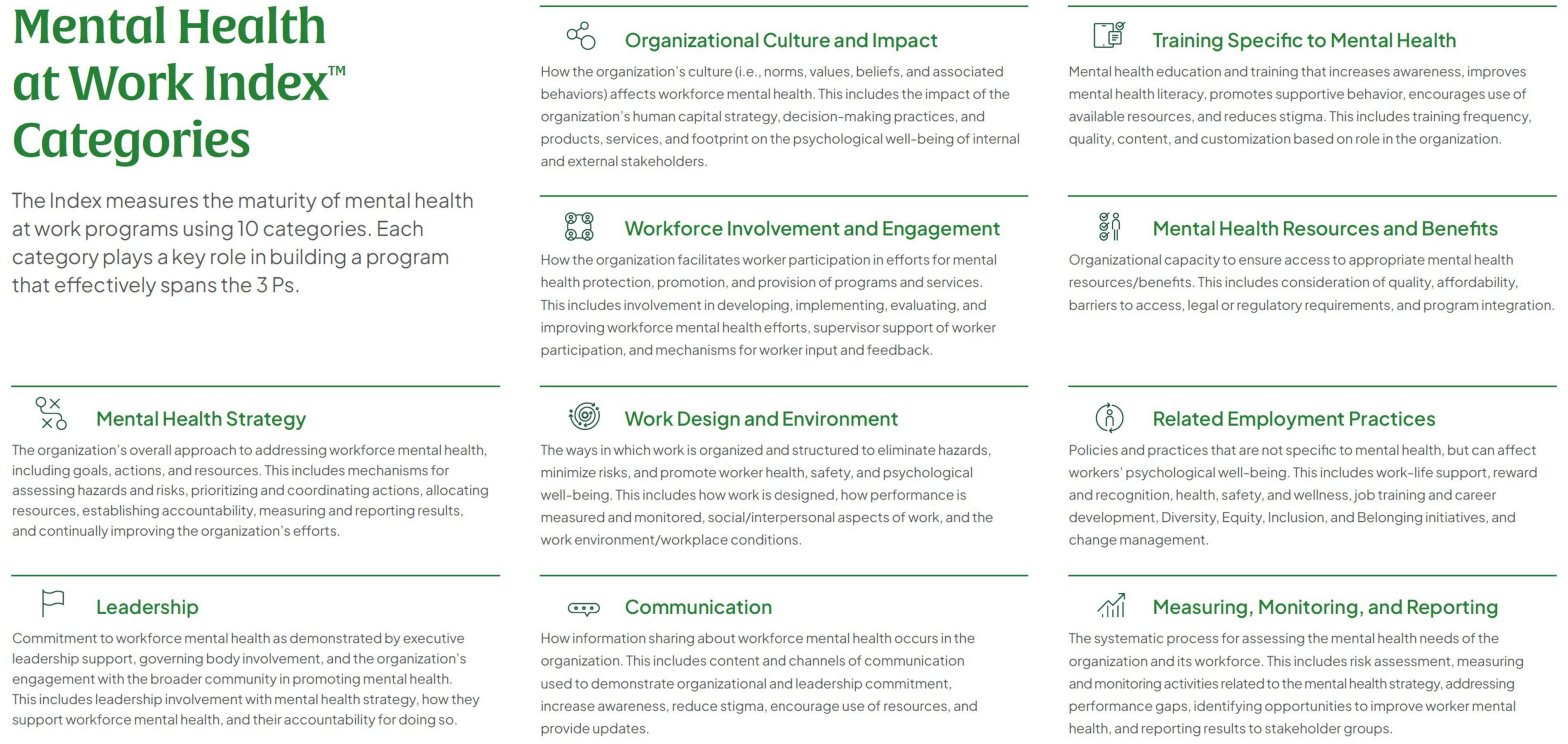
The mental health at work index 10 categories of organizational-level workforce mental health practices.

The 3 Ps—protection, promotion, and provision—apply to the 10 categories of organizational practices that support and advance workforce mental health: mental health strategy; leadership; organizational culture and impact; workforce involvement and engagement; work design and environment; communication; training specific to mental health; mental health resources and benefits; related employment practices; and measuring, monitoring, and reporting. A brief description of each category is provided in Figure 2.

### 4.1 Mental health strategy

Strategic planning is “a deliberative, disciplined effort to produce fundamental decisions and actions that shape and guide what an organization (or other entity) is, what it does, and why” (36). The strategic planning process generates a framework for setting goals, guiding decisions, and allocating resources. Although strategic planning is often thought of as an organizational-level effort, the process can also be applied to specific intra-organizational functions and initiatives and has been identified as a best practice element in the leading industry assessments of worksite health and well-being programs (28, 37, 38). An explicit workforce mental health strategy can similarly help guide comprehensive evidence-based actions for optimal results (39).

The Mental Health Strategy category reflects an organization’s overall approach to addressing workforce mental health, including goals, actions, and resources. This encompasses mechanisms for assessing hazards and risks, prioritizing and coordinating actions, allocating resources, establishing accountability, measuring and reporting results, and continually improving the organization’s efforts. In addition to outlining these process steps, a formal strategic plan for workforce mental health details the roles, responsibilities, and actions related to key stakeholder groups (e.g., senior leaders, managers, employees, governance bodies, union representatives). The breadth of stakeholders highlights the value of an inclusive strategic planning process that combines diverse perspectives of a cross-functional team and uses input from workers across the organization to inform the strategy.

The strategic plan for a mental health program includes how the organization will address the full range of needs for its entire workforce population. This spans the 3 Ps, protecting worker mental health by eliminating hazards and reducing risks, promoting positive mental health and psychological well-being, and providing access to benefits and resources that help people manage both acute and chronic mental health concerns. Mature plans integrate with the organization’s overarching strategic and operational goals, align with related policies and practices, and coordinate with diversity, equity, inclusion, and belonging initiatives in pursuit of common goals. A strategic plan is a living document. As such, it should include mechanisms for ongoing review, evaluation, and improvement.

### 4.2 Leadership

In the complex landscape of organizational dynamics, leadership is an influential force that can either foster or hinder the well-being of workers (40). The interplay of leader behaviors, the quality of leader-employee relationships, and specific leadership styles have a profound impact on worker stress, affective well-being, and overall workplace productivity (41). While poor leadership is well documented to have detrimental effects on workers (42), effective leadership has been linked to positive outcomes. A meta-analysis by Kuoppala et al. (43) found moderately strong relationships between leadership and mental health outcomes such as anxiety, depression, and work stress.

The Leadership category covers leaders’ commitment to workforce mental health, including executive leadership support, governing body/board involvement, and the organization’s engagement with the broader community and public in promoting mental health. This includes leadership involvement with the organization’s mental health strategy, how they advance and support workforce mental health, and the accountability mechanisms in place for doing so.

Through their words and actions, leaders can help advance and support an organization’s workforce mental health efforts in a variety of ways. By establishing mental health as a priority and regularly communicating with workers about it, leaders convey the organization’s commitment to the psychological well-being of the people who work there. An even more powerful message is shared when leaders actively participate in mental health initiatives themselves, take steps to address workplace issues that affect workforce mental health, and hold direct reports accountable for doing the same in their respective business units. In the most mature organizations, executive leaders may have formal performance goals and expectations related to advancing workforce mental health, and engage in public and industry thought leadership on the topic.

Having one or more senior leaders serve as an executive sponsor for an organization’s workforce mental health efforts can help ensure top level oversight and keep the issue at the forefront for the leadership team (20, 44, 45). This can be further strengthened by making mental health a shared responsibility between executive leadership and governance members. Providing the board of directors or other oversight body with regular updates (e.g., changes to mental health resources and benefits, outcomes of programs and initiatives, assessment of psychosocial hazards and risks, relevant complaints and resolutions, employee survey results) helps members make informed decisions in upholding the duty of care that is part of their fiduciary responsibilities, links mental health to workforce sustainability and organizational performance, and fosters accountability by encouraging executive leadership to advance mental health. Organizations with mature programs may even include workforce mental health expertise on the skills matrix for their governing body and/or designate a seat for a member who has formal responsibilities in this area and makes the topic a recurring part of the agenda.

### 4.3 Organizational culture and impact

Culture is the set of shared assumptions that guide behavior in an organization (46). Those norms, values, and beliefs function at multiple levels and are communicated to people when they are hired and are reinforced over time as the correct way of doing things (47, 48). In recent years, workplace health promotion scholars and practitioners alike have emphasized the importance of going beyond wellness programs and initiatives that focus on the individual to build a culture of health; an environment where worker well-being is valued and reflected in all aspects of how an organization operates (49–52).

The Organizational Culture and Impact category focuses on the ways in which an organization’s culture affects workforce mental health. This includes how mental health considerations are built into the organization’s human capital strategy (e.g., hiring, selection, promotion, accommodations practices), how organizational decision-making affects workforce psychological well-being, aspects of the organization’s culture that are tied to psychosocial hazards and risks, and the psychological impact of the organization’s products, services, and footprint on internal and external stakeholders.

An organization’s commitment to mental health can be reinforced by leader communications, articulated in materials like values statements and annual reports, and reflected in actions throughout the organization, such as how people treat each other and the mental health impact of decision-making processes. Concrete actions to promote a positive social climate play a significant role in supporting workforce mental health. This includes norms and behaviors related to how people in the organization interact with and relate to one another such as civility, trust, social support, and psychological safety (53–60).

Organizations with the most mature workforce mental health programs take steps to align their other organizational policies and practices with their mental health strategy. This could include adjusting hiring, training, scheduling, time off, and performance management practices to reduce unnecessary stressors. Other actions can include explicitly incorporating mental health in ESG reporting frameworks and integrating workforce mental health programs with ethics, corporate social responsibility, and diversity, equity, inclusion, and belonging initiatives. Similar to what has become common practice in corporate ethics, some organizations are expanding their influence, holding third parties accountable for abiding by the same standards and considering mental health practices when selecting vendors, suppliers, and partners. In the broadest sense, organizations can also periodically assess the mental health impact of their products, services, and footprint, take steps to both eliminate hazards and contribute positively to the psychological well-being of internal and external stakeholders, and engage with partners to address industry-level impact on society.

### 4.4 Workforce involvement and engagement

Decades of research links high-involvement management practices to worker well-being and organizational performance (61–65). A key element of the ISO guidance on psychological health and safety efforts at work (20) is consulting with and engaging workers throughout the entire process of designing, implementing, evaluating, and improving programs and policies, which can increase workers’ commitment and motivation to contribute to a healthy work environment. Doing so has been linked to reducing psychosocial risks and improving worker mental health (66) and has the added benefit of helping an organization put initiatives in place that are tailored to meet the unique needs of its workforce and, therefore, more likely to be successful (67).

The Workforce Involvement and Engagement category considers how an organization facilitates worker participation in its mental health efforts. This includes supervisor support of worker participation, and mechanisms for soliciting worker input and feedback. Two key involvement practices are establishing a cross-functional team that has direct input into the organization’s workforce mental health efforts and developing a network of champions who serve as local ambassadors in their respective business units. Having a cross-functional team of workers from various departments, units, or job functions who are collectively responsible for the organization’s mental health efforts brings diverse perspectives to the table and allows for information and resources to be shared across the organization. A champions network can further broaden participation in organizational mental health efforts by tapping grassroots influencers to help increase awareness, share communications, encourage participation among their peers and colleagues, and direct people to available mental health resources when needed.

Supervisors have a significant impact on workers’ day-to-day experience on the job and quality of work life (68, 69). Research suggests that supervisor support encourages participation in workplace wellness programs (70) and managers can engage their direct reports in a variety of ways. From highlighting mental health messages and tailoring team-specific communications to soliciting input and feedback and sharing it up the reporting line, supervisors are an important conduit for information. Managers’ actions, such as encouraging worker participation in mental health efforts, carving out work time for them to do so, recognizing team members who are contributing to the efforts, and visibly participating themselves are critically important. These actions model the desired behaviors and demonstrate that supervisors also see workforce mental health as a priority.

There are many ways to collect worker input and feedback related to workforce mental health, and mature efforts use multiple mechanisms that align with worker communication preferences. These can be formal and informal, in-person and technology-facilitated, solicited and unsolicited, and individual and group opportunities. Examples include workforce surveys, all-hands meetings, one-on-one or small group interactions with managers and leadership, physical and virtual suggestion boxes, and anonymous whistleblower hotlines. Collecting input and feedback from workers is not enough, however. Organizations must put that information into action and relay back to workers how their input was used (71–75).

### 4.5 Work design and environment

The way work is organized and conducted can affect both worker well-being and organizational functioning. Work design, interpersonal interactions, and the work environment itself can expose workers to psychosocial hazards that pose risks to their mental health (76) and create liabilities for the organization in terms of absenteeism, turnover, hiring and training costs, lawsuits and complaints, and reputational damage (20). Conversely, healthy work design can positively affect workers’ well-being, satisfaction, and functioning in both their work and non-work lives and help increase work engagement, productivity, innovation, and workforce sustainability (20, 77).

To protect workers from harm and promote the positive impact of work, employers need to manage a variety of factors. This spans: work design issues such as autonomy and control (78), job demands (e.g., workload and hours) (79), and job insecurity (80); interpersonal relations including social support (81), conflict (82), and mistreatment (83, 84); and elements of the physical work environment like temperature, lighting, and air quality (85–87). The Work Design and Environment category of the Mental Health at Work Index Framework is informed by and closely aligned with ISO’s international guidelines for managing psychosocial risks at work (20). These guidelines apply an occupational health and safety management systems approach to identifying psychosocial hazards, assessing the risks they pose, and managing those risks. Examples of the hazards are listed in Table 1, based on ISO 45003: Guidelines for Managing Psychosocial Risks (20).

**Table 1:** ISO 45003 identification of psychosocial hazards.

Hazard	Examples
How work is designed	
Roles and expectations	Ambiguity Conflict Performing work of minimal value
Job control or autonomy	Limited input into decision making Lack of influence over work tasks and workload Minimal independence in how work is performed
Job demands	Conflicting deadlines Underuse of skills High levels of interaction with others
Organizational change management	Prolonged or recurring change Lack of consultation or communication Insufficient support during transitions
Remote and isolated work	Isolation Lack of social support or contact Working in remote or private locations
Workload and work pace	Overload or underload Time pressure Deadlines
Working hours and schedule	Shiftwork Inflexible schedules Unpredictable hours
Job security and precarious work	Risk of redundancy Non-standard employment Lack of labor law protection
Social factors	
Interpersonal relationships	Poor communication Interpersonal conflict Power differentials between groups of workers
Leadership	Management style Lack of accountability Abuse of power
Organizational and workgroup culture	Lack of support for personal development Lack of alignment on objectives Uneven application of policies and procedures
Recognition and reward	Effort-reward imbalance Lack of appreciation Unfair distribution of rewards and recognition
Career development	Under-promotion Lack of opportunities for skill development Minimal or uncertain career opportunities
Support	Inadequate supervisor support Lack of information about work performance Limited access to resources
Supervision	Inadequate performance feedback Lack of fairness Misuse of performance surveillance
Civility and respect	Lack of trust Dishonesty or unfairness Inconsiderate interactions
Work-life balance	Spillover into personal time Conflicting demands Lack of recovery time
Violence at work	Abuse Threats Assault
Harassment	Offensive, unwanted or intimidating behavior based on personal characteristics Based on race, gender, or sexual orientation Based on religion, disability, or age
Bullying and victimization	Isolation Intimidation Undermining or sabotage
Work environment, equipment, and hazardous tasks	
Equipment	Unreliable In disrepair
Workplace conditions	Poor lighting Excessive noise
Availability of necessary tools, equipment, and resources	Lack of tools and equipment Insufficient resources to complete work tasks
Extreme working conditions	High or low temperatures Working at height
Working in unstable environments	Working in conflict zones Exposure to hazardous situations

Based on ISO 45003: Guidelines for Managing Psychosocial Risks (20).

Controlling and minimizing exposure to psychosocial hazards is key to reducing the risk of negative consequences for worker well-being and organizational functioning. Protecting workers’ mental health and promoting psychological well-being requires a multi-level approach that includes organizational and job-level changes, as well as individual-focused efforts (88, 89). The hierarchy of controls, a common occupational safety and health framework for determining how to best reduce hazard exposures, can be modified and applied to psychosocial hazards (30, 90). The various approaches are typically presented in descending order as an inverted pyramid with the most effective and reliable approaches (i.e., changing the work environment and work practices to eliminate hazards and reduce risks) at the top. While many workplace health promotion and wellness efforts focus heavily on encouraging individual-level behavior change and educating workers, those approaches actually sit at the bottom of the pyramid as the least effective strategies.

Mature workforce mental health efforts have processes for proactively identifying psychosocial hazards and managing risks, policies and practices that promote psychological well-being, and work design that bolsters mental health. Sophisticated workforce mental health programs go beyond considering the hazards and risks generally present in an organization to also identify and address those that specific worker segments (e.g., by job, function, location, or certain demographic characteristics) or individuals with different working arrangements (e.g., onsite, remote, hybrid) may be exposed to.

### 4.6 Communication

Communication is an essential part of any effort to promote workforce health and well-being and is critical to success (91). Communications related to workforce mental health serve to make workers aware of available benefits and resources and how to access them, demonstrate leadership’s commitment and support, and provide timely, relevant content to various stakeholder groups. Additionally, effective workforce mental health communications can help reduce stigma and improve employee help seeking (92). Practices in the Communication category include having a formal communication plan for workforce mental health efforts that is aligned with an overarching mental health strategy, implemented consistently, and updated regularly based on changing risks and needs. An effective communication plan details the various stakeholder groups (e.g., workers, family members, governance bodies, supervisors, owners/investors) who will receive targeted, relevant messages, the content of those communications, and the delivery mechanisms employed. Tailored communications are important to ensure that messaging resonates with an organization’s intended audience and ultimately reaches them through the channels selected (93, 94). In effect, the organization needs to reach the right audiences with the right messages using the most effective communication channels.

In mature communication efforts, content shared with stakeholders spans the 3 Ps, protection of mental health (e.g., information about psychosocial hazards and risks, normalizing talking about mental health in order to reduce stigma), promotion of psychological well-being (e.g., self-care tips, commitment from leaders, progress toward organizational mental health goals), and provision of access to information, resources, and services (e.g., details about how to access the EAP, changes to behavioral health benefits, crisis management plans).

Cutting through the clutter of today’s information-dense world to get people’s attention and craft messages that stick depends on the effective deployment of multi-channel communication. Fortunately, there are a variety of tools available to support mental health communications in the work setting. This may include print and electronic communications (e.g., company newsletters, emails, brochures, digital signage, intranet pages, home mailings, messaging and team collaboration platforms), in-person or virtual meetings (e.g., all hands, town halls, department meetings, one-on-one exchanges between workers and supervisors), and other programs and initiatives (e.g., mental health month activities, podcasts and videos, presentations from health and wellness staff or vendors). Worker input and feedback about messaging and channels can help increase the impact of mental health communications, as can regularly evaluating whether messages are reaching the relevant audiences and driving intended outcomes.

### 4.7 Training specific to mental health

Training related to mental health has gained in popularity in recent years and is often a core element of employers’ workforce mental health efforts. It is important, however, to understand the benefits and limitations of such training, to implement evidence-based programs as part of a comprehensive mental health strategy, and to evaluate the effectiveness of training efforts over time. These steps not only help to ensure that training participants are achieving the desired learning outcomes, but also verify that resources allocated to training programs are being well spent.

Research suggests that awareness and literacy training can help increase individuals’ knowledge about mental health issues, improve attitudes, encourage help seeking, and reduce stigma, at least in the short run (92), although additional follow-up training may be necessary to sustain results over time (95). The WHO guidelines on mental health at work (6) conditionally recommend mental health training for workers as a way of improving mental health literacy, awareness, and attitudes and reducing stigmatizing attitudes. One of the strongest recommendations in the WHO guidelines (6) is to train managers to support worker mental health in order to improve supervisors’ knowledge, attitudes, and behaviors and to encourage workers to seek help when needed. Broader manager training can also help supervisors foster better relationships with workers, identify and address psychosocial stressors, and design healthy work environments that are conducive to mental health and psychological well-being.

The Training Specific to Mental Health category covers an organization’s mental health education and training activities, including the frequency, quality, and content offered, as well as which organizational stakeholders receive training and how materials are customized to meet their specific needs. Mature training efforts are designed to support an organization’s mental health strategy and are delivered on an ongoing basis using materials that are developed by experts and updated regularly. In addition to employees and managers, comprehensive training efforts can include tailored programming for senior leaders, board and governance members, contractors and vendors, industry partners, and groups with responsibilities related to workforce mental health (e.g., cross functional workgroups, champions network). Training content can cover a range of topics that include mental health literacy, available resources and benefits and how to access them, and crisis management related to critical incidents such as disasters and workplace violence. Comprehensive efforts go well beyond basic awareness, however, to include information about risk-reduction strategies and organizational practices that support psychological well-being.

Well-designed mental health training also includes robust evaluation mechanisms to determine whether the desired outcomes are being achieved and training resources invested wisely. In addition to trainee satisfaction and whether learning objectives were met, sophisticated evaluation methods will also include whether the knowledge and attitudinal shifts are retained over time and if the desired behavioral changes (e.g., more help seeking and support behaviors, increased use of cost-effective benefits and resources, application of healthy work design principles) are taking place as a result. Regular evaluation of training, especially regarding behavior and outcomes, promotes increased accountability for training results and provides valuable information that can be used for continual improvement of the training program moving forward (96).

### 4.8 Mental health resources and benefits

Mental health benefits provide coverage for assessment and treatment of mental health and substance use disorders. There are various healthcare models across the globe, with service delivery and funding coming from government, private organizations, or a combination of the two. When funding is private, employers may pay for or subsidize care and in circumstances when services are covered by the government (i.e., funded through taxes), employers may provide supplemental insurance or private care outside of the government health system. Regardless of which model is in place, organizations benefit from supporting workers in accessing necessary services, so they remain healthy and performing at their best. Minimum standards may apply and vary depending on jurisdiction. Therefore, employers must ensure that any mental health resources and benefits they provide meet the legal and regulatory requirements in the jurisdictions in which they operate. Beyond meeting minimum standards, however, effective solutions ensure workers have equitable access to high-quality, affordable services that meet their needs in a timely manner.

Mental health benefits span a wide range of clinical services, including both inpatient treatment and outpatient psychotherapy, which are important for worker mental health and productivity (97, 98). Outpatient treatment is increasingly available through telehealth services and other technology-enabled delivery mechanisms, which reduce barriers to access and make mental health services more convenient in terms of both time and location (99). Employee Assistance Programs (EAPs), an employer-funded benefit that provides access to short-term counseling designed to promote healthy functioning in the workplace at no cost to workers, is another common offering that can support workforce mental health. Milot (100) found that EAP users had significantly reduced psychological distress and work presenteeism, as well as increased work engagement and life satisfaction, when compared to non-EAP users. Similarly, a meta-analysis by Chen et al. (101) found that EAPs are linked to lower work stress and higher levels of job satisfaction, commitment to the organization, and social support. Additionally, more than a decade of research on users of EAP services found significant improvements in absenteeism, presenteeism, work-related distress, employee engagement, and life satisfaction (102).

Beyond benefits covering mental health treatment, providing resources such as workshops or wellness programs focused on mindfulness, meditation, or similar self-care practices have also been found to be effective in improving worker psychological well-being. Other examples include mental health support groups, informational and self-help materials, return to work programs following mental health leave, support in navigating mental health benefits and resources (e.g., healthcare concierge or benefits advisor), and accommodations related to mental health issues.

The Mental Health Benefits and Resources category considers the range of benefits and resources available to workers, as well as their quality, affordability, accessibility, and extension to family members. The range of mental health and substance use disorder benefits that workers have access to will impact the extent to which an organization can meet the diverse needs of stakeholders. Ensuring access to a wide range of benefits (e.g., preventive care, diagnostic assessment, outpatient psychotherapy, inpatient services, substance use/addiction treatment) will increase the likelihood that workers will have the support they need to address mental health and substance use issues for themselves and their family members, and remain productively engaged in their work (103–106).

Accessibility and affordability are also important to consider, as they often pose barriers to workers obtaining mental health benefits and resources even when available. Mature efforts ensure equitable access to a broad network of providers, timely appointments, affordable services with no unreasonable limits, and culturally appropriate care that is coordinated and integrated across service providers for a seamless experience. It is critical that organizations attempt to minimize barriers to access because doing so allows for improvements in employee outcomes and organizational performance (107). Employers should periodically review and take steps to improve mental health benefits and resources based on outcomes, utilization, and stakeholder input. Monitoring mental health trends and regularly collecting feedback on mental health offerings allows organizations to identify areas of improvement and obtain an accurate picture of how well their mental health resources and benefits are meeting workforce needs.

### 4.9 Related employment practices

There are a variety of organizational policies and practices that are not specific to mental health but can affect workers’ psychological well-being. This includes work-life support, reward and recognition, health, safety, and wellness, job training and career development, Diversity, Equity, Inclusion, and Belonging (DEIB) initiatives, and change management. Collectively, these practices can help support an organization’s mental health strategy.

Conflict between work and non-work demands can negatively affect worker well-being on and off the job and impair workers’ functioning both at home and at work (108–112). Work-life conflict has been linked with adverse outcomes for workers, their families, and their employers (113). Potential negative consequences include job stress, burnout, and decreased job and overall life satisfaction, as well as higher rates of absenteeism and turnover intent (114–116). Efforts to promote work–life harmony can increase job satisfaction and productivity, strengthen commitment to the organization, and reduce absenteeism and turnover (117–119). Work-life support efforts include remote and hybrid work options, flexible scheduling, compressed work weeks, sufficient time off, caregiver resources, and processes to help people return to work following leave.

Mental health and physical health are closely linked, and effective workplace health promotion and wellness programs address both. When organizations take a holistic approach to well-being, they are likely to achieve better health outcomes overall. Investing in worker health and safety has been linked to increased productivity and reductions in health care costs, absenteeism, and injury rates (120–122). Examples of health and wellness initiatives include educational sessions and informational resources on health topics such as diet, nutrition, and healthy sleep, programs to help workers manage chronic health conditions, fitness center access, and workspace ergonomic assessments and adjustments (123–127). Making health and wellness resources available to workers’ spouses, domestic partners, and dependents can further improve health outcomes (128–130).

Opportunities for training and development help workers build new knowledge, skills, and abilities and apply them to their work (131). Gaining new skills and work experiences can enhance motivation and job satisfaction and help workers manage job stress more effectively (132, 133). In addition to developing job skills, training for a high-performing workforce includes a wide range of topics, such as management and leadership skills, “soft skills” (e.g., communication, interpersonal skills), and fostering a fair workplace that fully engages people from all backgrounds (134, 135). Career development resources (e.g., career counseling, clearly-defined career paths), continuing education support (e.g., subsidized continuing education courses, tuition reimbursement), and coaching or mentoring programs are additional ways of creating a culture that supports ongoing training and development (136).

Recognition mechanisms, which can be formal or informal, monetary or non-monetary, reward workers for their contributions to the organization. Acknowledging workers and making them feel valued has been linked to increased job satisfaction, morale, and self-esteem, as well as reductions in stress and emotional exhaustion (133, 137). Examples include fair and equitable compensation, promotions, verbal and written acknowledgement from supervisors, awards, celebratory events and activities, and peer recognition programs.

A body of empirical evidence consistently demonstrates that diversity, equity, inclusion, and belonging (DEIB) have a positive influence on various organizational outcomes. These encompass heightened levels of innovation, improved performance, broader engagement with diverse client bases, increased job satisfaction and organizational commitment, and a greater likelihood of employees remaining with the organization (138). When poorly managed, aspects of an organization’s diversity climate can also have negative effects on workers of color, including lower job satisfaction and organizational commitment and higher levels of cynicism and turnover intentions (139). Change management practices have the potential to enhance or diminish workforce mental health. Changes have the potential to affect psychosocial risks and even create new ones for workers (20). When organizations engage in effective change management practices, they can reduce the negative mental health effects on individuals and teams within the organization, as well as increase the likelihood that change is successful (140). This includes communicating clearly about the changes, setting concrete goals, explaining the rationale, providing opportunities for input and feedback from workers, ensuring supervisor support, and taking steps to monitor and address any adverse impacts on workers.

Aligning performance management practices with an organization’s mental health strategy could include, for example, explicitly integrating support for workers’ mental health into managers’ performance goals and taking corrective actions when employees behave in ways that negatively impact the mental health and psychological well-being of others. Additionally, having a formal process for identifying mental health risks posed by performance management practices (e.g., unnecessarily stressful promotion reviews) can help an organization take proactive steps to both reduce any potential negative impact and promote psychological well-being.

### 4.10 Measuring, monitoring, and reporting

To strengthen workforce mental health, organizations must not only develop a comprehensive strategy, but also build in mechanisms for ensuring effectiveness, accountability, and adaptability to evolving challenges. Measuring, monitoring, and reporting practices play a critical role in that regard. Without such systems in place, organizations are at risk for expending significant resources based on faulty assumptions and without knowing the impact of their efforts. A systematic approach starts with a baseline evaluation of the mental health needs of the organization and its workforce and also includes process and outcomes measures that assess the fidelity of implementation and whether initiatives are producing the desired results (141–143). Comprehensive measuring, monitoring, and reporting processes help an organization identify needs and risks, set priorities, establish measurable goals, make data-driven decisions, evaluate whether programs and policies are working, track trends, and improve their programs over time. By reporting results to appropriate stakeholders (e.g., workers, executive leadership, managers, governing body), an organization demonstrates its commitment to workforce mental health and reinforces a culture of openness, transparency, growth, and continual improvement (144).

The Measuring, Monitoring, and Reporting category takes a broad look at how an organization uses data to drive decision making for lasting impact on workforce mental health. This includes whether an organization gathers data about workers’ mental health and how it protects privacy and confidentiality of that information, if it has a systematic process for evaluating the effectiveness of its mental health efforts, what data sources are available (e.g., healthcare claims data, EAP utilization and outcomes, participation rates, clinical screening measures, risk assessments, workforce surveys), who results are reported to (e.g., senior leaders, supervisors, workers, board/governance group), whether measurement spans protection, promotion, and provision activities, and how data are used (e.g., to identify hazards, to establish priorities, to set goals, to monitor progress, to improve efforts over time).

Organizations with mature workforce mental health efforts also consider the impact of their products and services on both workers and external stakeholders (e.g., communities they serve, the general public). For example, workers in an organization that provides a service that benefits their community may feel a sense of pride and experience their work as being meaningful. Alternatively, people who work at an organization that sells products seen as harmful to the public may feel shame, worry what their friends and family might think, or even be subjected to public criticism.

Worker well-being and organizational functioning are closely linked. Therefore, organizations that effectively address the mental health needs of their workforce have the potential to positively impact other organizational outcomes, including recruitment and retention, performance and productivity, organizational reputation, and health-care costs (145, 146). Employers that understand the link between workforce mental health and performance track organizational performance indicators (e.g., turnover, job satisfaction, work engagement, employee net promoter scores, burnout), so they can monitor trends in these areas that may correspond to increased program maturity as they enhance their workforce mental health efforts over time.

## 5 Application of the mental health at work index framework and future directions

Based on the framework described above, a Mental Health at Work Index self-assessment was developed that spans the 3 Ps and 10 categories of practices. Employers rate the maturity of their efforts in each area and report a variety of descriptive items about the organization (e.g., size, region, industry) and a series of performance indicators (e.g., voluntary turnover, work engagement, employee likelihood to recommend). This provides a holistic view of how the organization is supporting workers’ mental health, a baseline assessment of program maturity, and a structured way to identify gaps where priority actions can be taken for improvement.

As the data set for the Mental Health at Work Index grows, it will provide valuable information to better understand the landscape of workforce mental health efforts across the globe, establish benchmarks for best practice programs, highlight areas of need, and drive the continued movement toward more effective practices. Developing a better understanding of the link between the maturity of mental health at work efforts and organizational performance indicators is a promising area to explore as more data become available. There may also be significant variations by industry, size, and geographic region that will require a larger data set to tease out. As more organizations complete the Index assessment, we have a longer-term opportunity to build an aggregated database of benchmarks and best-in-class workforce mental health programs that are targeted to these specific areas and provide even more relevant and actionable information.

## 6 Discussion

The Mental Health at Work Index provides an evidence-based framework that employers can use to assess their workforce mental health efforts and improve their program maturity, ultimately ensuring that practices are effective and resources are spent wisely. There are, however, some limitations based on the current state of knowledge. Our review of the literature related to workplace mental health efforts highlighted significant research gaps, including an overemphasis on narrow, individual-focused interventions rather than comprehensive, multi-component approaches. There is little research that evaluates complex, systemic workforce mental health efforts (147, 148). As a result, the Mental Health at Work Index Framework does not currently reflect relationships among the practice categories. As more organizations apply the framework, additional analyses will help to illuminate how various policies and practices relate to one another, as well as to employee and organizational outcomes. There would be great value in the development of additional studies focused on comprehensive, real-world programs. To this end, Rugulies et al. (149) suggest that the updated Medical Research Council guidance for developing and evaluating complex interventions (150) might be useful in studying comprehensive workforce mental health efforts, since multiple outcomes and contextual factors can be included.

There are also significant shortcomings on the practice side of the equation, such as the absence of clear strategies, proclivity to provide reactive, *ad hoc* solutions, little use of evidence-informed practices, lack of measurement, and limited efforts in healthy work design and the protection of workers’ mental health. In sum, opportunities are ripe for researchers and practitioners to work together to conduct high-quality research, translate results for application in real-world organizational settings, and improve the quality and effectiveness of workforce mental health efforts worldwide.

## 7 Conclusion

A growing number of employers recognize the need to prioritize workforce mental health for both the well-being of people who work there and the effective functioning of the organization itself. The business case is strong and complementary to the ethical and human imperative for organizations to create a mentally healthy workplace. Increasing the impact of mental health at work efforts, however, will require a comprehensive approach that protects worker mental health, promotes psychological well-being, and provides access to information, resources, and services, as needed.

In this article, we reviewed the range of evidence-informed actions needed for organizations to comprehensively address workforce mental health. The 3 Ps and 10 categories of practices that form the structure of the Mental Health at Work Index Framework and related assessment are intended to help employers measure and improve the maturity of their workforce mental health programs. By investing in a strategic, evidence-informed approach, organizations have a tremendous opportunity to systemically address workforce mental health and create work environments where individuals and organizations thrive.

## Footnotes

^1^ https://mentalhealthindex.org
